# Influence of sire fertility on the metabolism of *in vitro* produced embryos

**DOI:** 10.1530/RAF-26-0028

**Published:** 2026-07-15

**Authors:** Quinn A Hoorn, Elena O’Callaghan, David A Kenny, Sean Fair, Patrick Lonergan, Constantine A Simintiras

**Affiliations:** ^1^ School of Agriculture and Food Science, University College Dublin, Dublin, Ireland; ^2^ Animal and Bioscience Department, Teagasc, Dunsany, County Meath, Ireland; ^3^ Laboratory of Animal Reproduction, Department of Biological Sciences, Bernal Institute, University of Limerick, Limerick, Ireland; ^4^ School of Animal Sciences, Agricultural Center, Louisiana State University, Baton Rouge, Louisiana, USA

**Keywords:** bull, sire, embryo, metabolomics, fertility

## Abstract

**Abstract:**

Bull field fertility varies greatly and is largely influenced by differences in early embryo survival rather than fertilization failure alone. We, therefore, investigated whether sire fertility status is reflected in the metabolomic profile of embryo-conditioned culture media. Six Holstein-Friesian bulls were selected from a population of 840 sires and classified using an adjusted sire fertility index. Mean (±SD) values were 4.27 ± 0.61% for highly fertile bulls (HF, *n* = 3) and −13.1 ± 1.85% for sub-fertile bulls (SF, *n* = 3). Cumulus–oocyte complexes were fertilized using frozen–thawed semen from individual bulls. On day 7 post-fertilization, grade 1 blastocysts (pools of *n* = 15 per bull; *n* = 3 replicates) were transferred to fresh culture medium for 24 h. On day 8, embryo-conditioned media from both fertility treatments and unconditioned media were collected, snap-frozen, and subjected to high-throughput untargeted metabolomic profiling. A total of 149 metabolites were detected. Unsupervised analysis revealed substantial overlap between HF and SF sire-derived embryo-conditioned media, while both differed from unconditioned media. The relative abundance of 30 metabolites was altered by embryo presence, irrespective of sire fertility. Direct comparison of HF vs SF sire-derived embryo-conditioned media identified only three differentially abundant metabolites. However, multivariate machine learning analysis identified a panel of eight N-acetylated amino acids, which discriminated embryos derived using semen from bulls of divergent fertility status with high accuracy (AUROC = 0.903). In conclusion, sire fertility status imparts a subtle but detectable metabolic imprint on the early embryo, which can be exploited to non-invasively discriminate embryos according to potential developmental competence.

**Lay summary:**

Reproductive efficiency is fundamental to productive and profitable cattle farming. Even bulls that pass routine fertility assessments can achieve very different pregnancy rates. Understanding the biological basis of this variation has important practical implications. We studied whether differences in early embryo metabolism might help explain why some bulls consistently outperform others in fertility. We created embryos in the laboratory using sperm from highly fertile and sub-fertile bulls. Then, we analyzed hundreds of small molecules that reflect metabolic activity in the fluid surrounding the embryo, and we detected very few differences between the two groups. However, using a machine learning approach, we identified a panel of eight molecules that distinguished embryos by sire fertility status with high accuracy. These findings suggest that a bull’s fertility leaves a subtle but detectable metabolic imprint on early embryos, with potential future applications in non-invasive embryo quality assessment.

## Introduction

Sire selection is a cornerstone of genetic improvement and reproductive efficiency in cattle production systems. Despite passing routine semen quality evaluations, a proportion of bulls used in artificial insemination (AI) consistently achieve suboptimal pregnancy rates under field conditions, compromising both farm productivity and profitability (Fair & Lonergan 2018). These reduced pregnancy outcomes reflect a combination of fertilization failure and embryonic loss prior to routine pregnancy diagnosis around day 30 (Diskin & Morris 2008, Reese *et al.* 2020). Although maternal, environmental, and management factors contribute to pregnancy loss, substantial and repeatable variation exists among individual bulls, indicating a significant paternal component (Franco *et al.* 2020). Elucidating the paternal determinants of pregnancy success is, therefore, critical for improving bovine reproductive efficiency.

Molecular differences between sperm from highly fertile (HF) and sub-fertile (SF) bulls have been characterized using transcriptomic (Feugang *et al.* 2010, Donnellan *et al.* 2022), proteomic (Peddinti *et al.* 2008, Rabaglino *et al.* 2022, Fernández-Montoro *et al.* 2025), epigenomic (de Oliveira *et al.* 2013, Kropp *et al.* 2017, Kutchy *et al.* 2018, Costes *et al.* 2024), and metabolomic (Menezes *et al.* 2019, Longobardi *et al.* 2020, Saraf *et al.* 2020, Hoorn *et al.* 2025) approaches. These studies have yielded candidate sperm-based biomarkers with potential utility for predicting fertility prior to semen distribution for AI. However, such approaches provide limited insights into how paternal factors influence downstream biological and molecular processes within the early embryo that may ultimately determine pregnancy establishment.

Direct associations between field fertility and a bull’s capacity to generate embryos (*in vitro* or *in vivo*) have also proven inconsistent, in part due to substantial inter-bull variation (Ortega *et al.* 2018, O’Callaghan *et al.* 2021). Nonetheless, it is plausible that sire-driven molecular factors carried by sperm at fertilization induce subtle but biologically meaningful changes in embryo physiology that influence subsequent survival and manifest as variations in field fertility. Data from mouse and human studies show that paternally inherited molecules, including miRNAs, can influence embryo quality and pregnancy establishment (Yuan *et al.* 2016, Isacson *et al.* 2025). In cattle, Pasquariello *et al.* (2024) similarly reported differences in blastocyst miRNA content between bulls of divergent fertility status. Collectively, these findings highlight the importance of interrogating paternally dictated early embryonic phenotypes.

Metabolomic profiling of embryo-conditioned culture media provides a non-invasive means of assessing embryo physiology and developmental competence. In cattle, this approach has been associated with developmental progression to the blastocyst stage (Perkel & Madan 2017, Nõmm *et al.* 2019, Tsopp *et al.* 2025) and with pregnancy establishment following embryo transfer (Gimeno *et al.* 2021, 2023). Given that metabolite exchange reflects integrated cellular activity, embryo-conditioned media may capture subtle functional differences in embryo metabolism arising from paternal effects.

Accordingly, this study tested the hypothesis that embryos derived from bulls with divergent field fertility exhibit distinct metabolic signatures detectable in conditioned culture media and that multivariate machine learning-based analysis of these metabolic profiles can retrospectively discriminate embryos according to sire fertility status.

## Materials and methods

### Bull selection

Frozen–thawed semen from six Holstein-Friesian bulls was used for *in vitro* fertilization (IVF). All ejaculates passed standard commercial quality control procedures. Field fertility data were obtained from the Irish Cattle Breeding Federation (ICBF) database, and the fertility indices were calculated as previously described (O’Callaghan *et al.* 2021). Briefly, fertility was retrospectively determined from calving outcomes and adjusted for biological and management-related confounders. The resulting adjusted sire fertility index was centered around zero.

From a reference population of 840 Holstein-Friesian bulls, each with ≥500 insemination records, three highly fertile (HF) and three sub-fertile (SF) bulls were selected. HF bulls had a mean (±SD) fertility index of 4.27 ± 0.61% and a mean of 57,556 insemination records per bull (range: 34,859 to 99,953). SF bulls had a mean (±SD) fertility index of −13.10 ± 1.85% and a mean of 913 insemination records (range: 519 to 1,479) (Supplementary Table 1 (see section on Supplementary materials given at the end of the article)). The lower number of inseminations is due to sub-fertile bulls not being used as widely once AI companies become aware of their fertility problems.

### *In vitro* embryo production and sample collection

Three independent replicates of *in vitro* embryo production were performed as described by O’Callaghan *et al.* (2021). In brief, immature cumulus–oocyte complexes (COCs) were aspirated from local abattoir-derived ovaries and matured for 24 h at 38.5°C in 5% CO_2_ in air. Following maturation, COCs were washed and randomly allocated to one of the six bulls for IVF (approximately 100 COCs per bull per replicate). Frozen–thawed semen from each bull was processed to obtain motile sperm and added at a final concentration of 1 × 10^6^ sperm·mL^−1^. Gametes were co-incubated for 20 h at 38.5°C in 5% CO_2_ in air.

Following fertilization, presumptive zygotes were denuded by vortexing, washed in phosphate-buffered saline (PBS), and cultured in 50 μL drops of pre-equilibrated synthetic oviduct fluid (SOF) under oil at 38.5°C in 5% CO_2_ and 5% O_2_ under N_2_. SOF was prepared as previously described (Holm *et al.* 1999) and supplemented with 3 mg·mL^−1^ bovine serum albumin from the same lot and batch (Sigma-Aldrich, USA, Source: A9647). On day 7 post-fertilization, grade 1 blastocysts (according to the International Embryo Technology Society (IETS) manual) were removed from culture drops and transferred in groups of 15 into fresh 500 μL culture wells for a further 24 h. On day 8, 400 μL of embryo-conditioned media were collected from each well, snap-frozen, and stored in liquid nitrogen. This yielded 18 embryo-conditioned medium samples in total, corresponding to six bulls evaluated across three independent embryo production runs, with one conditioned medium sample generated per bull per run. Unconditioned control medium, incubated identically but without embryos, was collected and frozen in parallel across the same runs (*n* = 4).

### Mass spectrometry

Untargeted metabolomic profiling was performed by Metabolon Inc. (USA) using their standardized ultra-high-performance liquid chromatography–tandem mass spectrometry (UPLC–MS/MS) platform, as previously described (Simintiras *et al.* 2021, 2022, González-Brusi *et al.* 2025). In brief, samples were analyzed using reverse-phase UPLC–MS/MS in both positive and negative electrospray ionization modes, as well as hydrophilic interaction chromatography (HILIC) UPLC–MS/MS.

Metabolites were identified by matching chromatographic feature retention times and mass-to-charge ratios (m/z) to entries in the Metabolon internal spectral library, with a mass accuracy tolerance of ±10 ppm. Relative metabolite abundance is reported as raw peak intensities (Supplementary Table 2). Technical standards were co-run at randomized intervals and served as quality controls to monitor extraction efficiency, instrument performance, and analytical drift. Instrument variability, assessed as the median relative standard deviation of internal standards added to each sample prior to injection, was 11%.

### Qualitative metabolomic analyses

For qualitative summaries, including the Venn diagram depicting the distribution of metabolites among treatment groups, analyses were performed directly on the raw metabolite matrix and restricted to metabolites detected in ≥25% of samples within each group. Similarly, the pie chart summarizing metabolite super-pathway distribution was generated using all detected metabolites, irrespective of relative concentrations.

### Semi-quantitative metabolomic analyses

Missing values were replaced with zero to avoid potential imputation-based inflation of low abundance signals, which could introduce bias in fold change estimation. Downstream semi-quantitative analyses were performed using MetaboAnalyst 6.0 (Pang *et al.* 2024). To reduce noise, metabolites were first filtered by interquartile range, using an 11% cutoff – corresponding to the median technical relative standard deviation – which removed 17 metabolites. Peak intensities for the remaining 132 metabolites were then median-normalized, log_10_ transformed, and Pareto-scaled (mean-centered and divided by the square root of the standard deviation for each variable).

#### Global analyses

Unsupervised debiased sparse partial correlation (DSPC) network analysis was performed using the MetaboAnalyst Network viewer. Topology view and node-neighbor scope settings were applied to visualize the primary network structure. Principal component analysis (PCA) was performed for unsupervised dimensionality reduction, and 95% confidence ellipses were derived using permutational multivariate analysis of variance (PERMANOVA) based on Euclidean distances in the space defined by the first two principal components. Sparse partial least squares discriminant analysis (sPLS-DA) was performed using five components and ten variables per component. Model performance was evaluated by five-fold cross-validation, testing an increasing number of components while holding the number of variables per component constant at 10. Hierarchical clustering and associated heatmap visualization were generated using Euclidean distance and Ward’s linkage on auto-scaled data. Boxplots of select metabolite normalized relative concentrations (NRCs) were exported directly from MetaboAnalyst. Boxplots display group medians (horizontal lines), interquartile ranges (boxes), data dispersion (whiskers extending to 1.5 times the interquartile range), individual sample values (points), and group means (yellow central rhombi).

#### Pairwise analyses

Pairwise comparisons were performed in MetaboAnalyst 6.0 using unpaired Student’s *t*-test assuming equal group variance. Corresponding *P*-values and fold changes are provided in Supplementary Table 3. Correlation heatmaps were generated using auto-scaled data and Pearson correlation to visualize clustering and the overall correlation structure among samples. Volcano plots were created in GraphPad Prism 10.5.0 (v. 673) for Mac using exported data (Supplementary Table 3).

For metabolite set enrichment analysis (MSEA), metabolite identifiers were harmonized within MetaboAnalyst 6.0 using the Human Metabolome Database (HMDB), PubChem, and the Kyoto Encyclopedia of Genes and Genomes (KEGG) databases. Features that could not be mapped to any of these resources were excluded from pathway-level analyses. MSEA was performed using the global test (Goeman *et al.* 2004) against the Relational Database of Metabolomics Pathways (RaMP-DB). Only metabolite sets containing at least two mapped metabolites were considered. Enrichment scores were calculated as the ratio of observed to expected metabolites within each pathway (Lu *et al.* 2023).

Pathway topology analysis was conducted separately and restricted to metabolites identified as differentially abundant (*P* ≤ 0.05). These metabolites were mapped to the KEGG *Bos taurus* library, and pathway impact was quantified using relative betweenness centrality. Resulting scatter plots depict enrichment significance (−log_10_ *P* value) vs pathway impact such that pathways containing altered, highly connected metabolites receive higher impact scores.

Together, enrichment and topology analyses provide complementary information: i) whether specific pathways are over-represented based on the number of altered metabolites (enrichment) and ii) the structural importance of those metabolites within each pathway (impact). Additional implementation details are available at metaboanalyst.ca.

#### Biomarker analysis

Biomarker analysis was performed in MetaboAnalyst 6.0, using its receiver operating characteristic (ROC) curve-based model evaluation framework, similarly to Absalon-Medina *et al.* (2025). In brief, pre-processing of raw peak intensities (filtering, normalization, transformation, and scaling) followed the workflow described above. Candidate metabolites were then manually selected for classification, and multivariate ROC models were built using the linear support vector machine algorithm. Model performance was estimated using 100-fold cross-validation, and mean outputs were used to generate ROC curves, 95% confidence intervals, and overall prediction accuracy. Empirical *P*-values were calculated from 100 permutations of the area under the ROC curve (AUROC).

## Results

**Figure 1:**
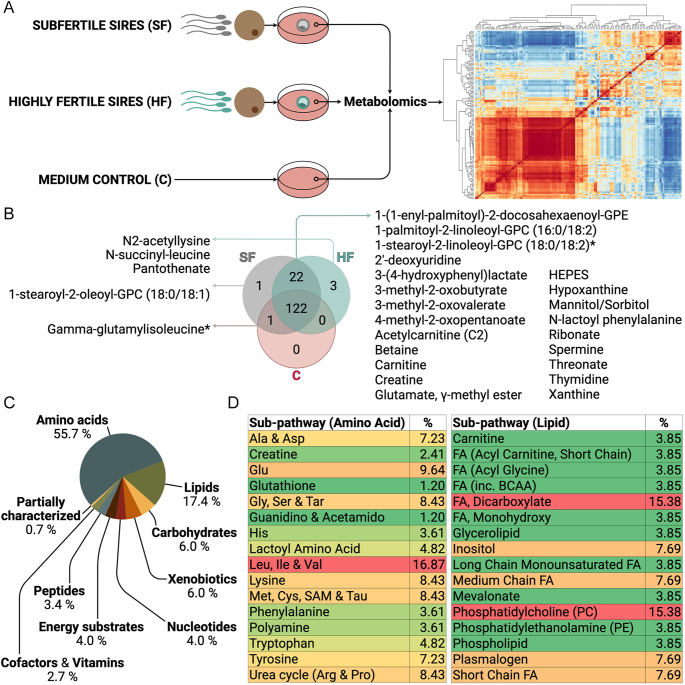
Overview of experimental design and metabolite classification. (A) Schematic depiction of the experimental groups used for untargeted metabolomic profiling of embryo culture media derived using semen from highly fertile (HF) and sub-fertile (SF) sires, alongside unconditioned medium controls (C). (B) Venn diagram and accompanying lists showing metabolites unique to, or shared between, each comparison. (C) Super-pathway distribution of all identified metabolites, expressed as the percentage of total identified biochemicals. (D) Sub-pathway composition within the amino acid (left) and lipid (right) super-pathways, presented as the proportion of total metabolites assigned to each sub-pathway. Colors indicate the relative proportion of detected metabolites within each sub-pathway, with red indicating a higher proportion, green indicating a lower proportion, and yellow denoting intermediate values.

Conditioned culture media from embryos derived from HF vs SF bulls, together with unconditioned (control) culture media, were subjected to untargeted metabolomic profiling to determine metabolite relative abundance (Fig. 1A). In total, 149 metabolites were detected, of which 122 were present in ≥25% of samples across all groups (Fig. 1B). Detected metabolites cluster within the following super-pathways: amino acids (55.7%), lipids (17.4%), xenobiotics (6%), carbohydrates (6%), nucleotides (4%), energy substrates (4%), peptides (3.4%), cofactors/vitamins (2.7%), and partially uncharacterized (0.7%) metabolites (Fig. 1C). Within the dominant super-pathways, amino acids were primarily associated with leucine, isoleucine, and valine metabolism (16.87%), whereas lipids were predominantly linked to dicarboxylate (15.38%) and phosphatidylcholine (15.38%) metabolism (Fig. 1D).

**Figure 2:**
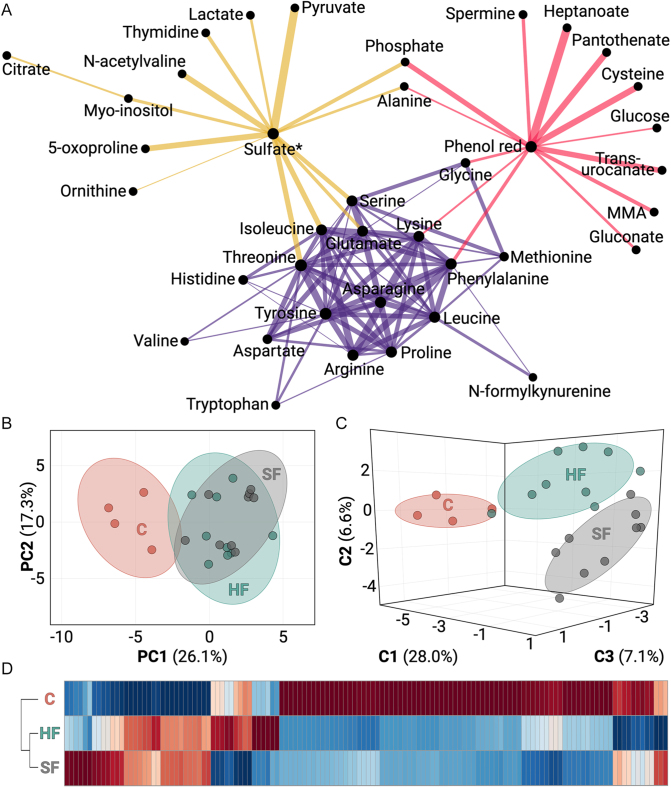
Multivariate analysis of the embryo culture media metabolome. (A) Debiased sparse partial correlation (DSPC) network of identified metabolites. The nodes represent individual metabolites, and the edge thickness reflects the strength of conditional associations. Colors indicate three major correlation-based clusters. (B) PCA, (C) sparse partial least squares discriminant analysis (sPLS-DA), and (D) hierarchical clustering of embryo culture media derived using semen from highly fertile (HF) and sub-fertile (SF) sires, alongside unconditioned medium controls (C).

Network analysis of the full dataset highlighted a dense correlation structure among amino acid-related metabolites (Fig. 2A). Unsupervised PCA revealed extensive overlap between embryo-conditioned media from HF and SF bull-derived embryos, indicating broadly similar embryo metabolism. By contrast, both embryo-conditioned groups separated clearly from unconditioned controls, as expected (Fig. 2B). Supervised sPLS-DA – a multivariate machine learning approach for separating potentially non-linear group structure (Lê Cao *et al.* 2011, Ruiz-Perez *et al.* 2020) – distinguished all three groups, confirming subtle differences between HF vs SF bull-derived embryo-conditioned media (Fig. 2C), which were not apparent by PCA. These patterns were broadly recapitulated by hierarchical clustering (Fig. 2D).

**Figure 3:**
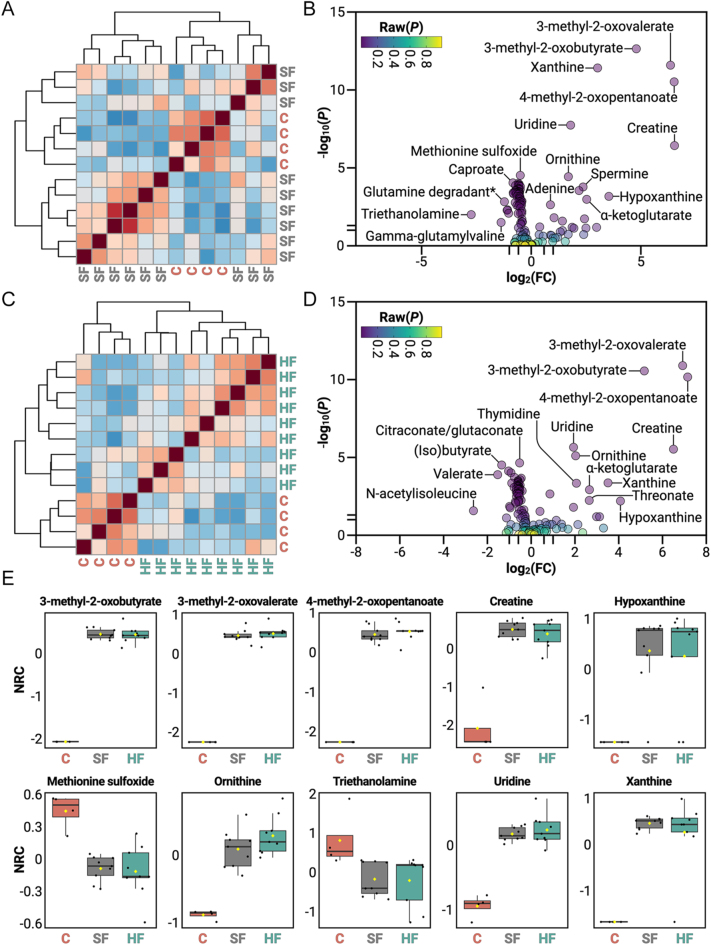
Differential metabolites in embryo-conditioned media vs control. (A) Correlation heatmap with hierarchical clustering of embryo culture media from sub-fertile (SF) sires vs unconditioned medium controls (C). (B) Volcano plot showing differentially abundant metabolites (DAMs) between SF and C samples. (C) Correlation heatmap with hierarchical clustering of embryo culture media from highly fertile (HF) sires vs C. (D) Volcano plot showing DAMs between HF and C samples. (E) Boxplots of NRCs of DAMs across C, HF, and SF groups.

Hierarchical clustering of SF vs control samples segregated the dataset into three clusters, comprising a homogenous control cluster and two SF subclusters with related but distinct profiles (Fig. 3A). Relative to the control, 15 metabolites were more abundant, whereas 23 were less abundant (*P* ≤ 0.05 and absolute fold change > 1.5) in SF bull-derived embryo-conditioned media (Fig. 3B). An additional 40 metabolites differed when significance was considered using *P* ≤ 0.05 alone, without applying the absolute fold change (|FC|) threshold. Moreover, following Benjamini–Hochberg false discovery rate (FDR) correction to adjust for multiple testing, these findings were largely preserved: 15 metabolites remained more abundant and 22 less abundant (*P* ≤ 0.05; |FC|>1.5), with only γ-glutamylvaline no longer meeting the combined criteria.

Hierarchical clustering of HF vs control samples similarly revealed group-level signatures (Fig. 3C). Compared with the control, 13 metabolites were increased and 31 were decreased in HF bull-derived embryo-conditioned media (*P* ≤ 0.05; |FC|>1.5) (Fig. 3D), with a further 28 metabolites differing at *P* ≤ 0.05 but below the FC threshold. Following FDR correction, these findings were similarly largely preserved: 13 metabolites remained more abundant and 29 less abundant (*P* ≤ 0.05; |FC|>1.5), with only azelate and triethanolamine no longer meeting the combined criteria. Boxplots of NRCs for the most affected metabolites across comparisons (SF and HF vs control) are shown in Fig. 3E. In total, 52 metabolite NRCs were altered in SF and HF bull-derived embryo-conditioned media vs unconditioned controls (*P* ≤ 0.05; |FC|>1.5), of which 30 were shared and changed in the same direction. A further 39 metabolites were affected when considering *P* ≤ 0.05 without the FC cutoff. FC and *P*-values for all metabolites are provided in Supplementary Table 3.

**Figure 4:**
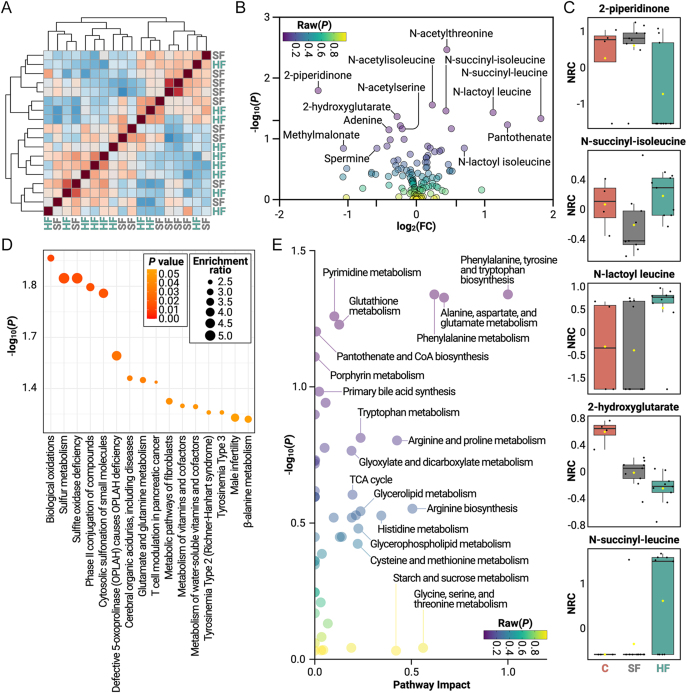
Comparative metabolomic profiles of embryo culture media conditioned by embryos derived from highly fertile (HF) and sub-fertile (SF) sires. (A) Correlation heatmap with hierarchical clustering of embryo culture media from SF vs HF. (B) Volcano plot showing differentially abundant metabolites (DAMs) between SF and HF samples. (C) Boxplots of NRCs of DAMs across SF, HF, and unconditioned medium control (C) groups. Pathway (D) enrichment and (E) impact analyses of DAMs between SF and HF groups.

Direct comparison of HF vs SF bull-derived embryo-conditioned media did not yield a clear separation by hierarchical clustering (Fig. 4A), consistent with broadly similar profiles observed by PCA. Differential abundance analysis identified only three metabolites (2-piperidinone, N-lactoyl leucine, and N-succinyl leucine) that met combined significance and FC criteria (*P* ≤ 0.05; |FC|>1.5) (Fig. 4B). Four additional metabolites (2-hydroxyglutarate, N-acetylisoleucine, N-acetylthreonine, and N-succinyl-isoleucine) differed at *P* ≤ 0.05 but did not meet the FC threshold. NRC distributions are shown in Fig. 4C. It is worth noting that no differentially abundant metabolites were detected in this comparison when FDR correction was applied.

Pathway enrichment analysis of these differentially abundant metabolites indicated enrichment of biological oxidations and sulfur metabolism, among other processes (Fig. 4D), while pathway topology analysis highlighted a notable impact across multiple amino acid metabolism pathways, including phenylalanine, tyrosine, and tryptophan biosynthesis (Fig. 4E).

**Figure 5:**
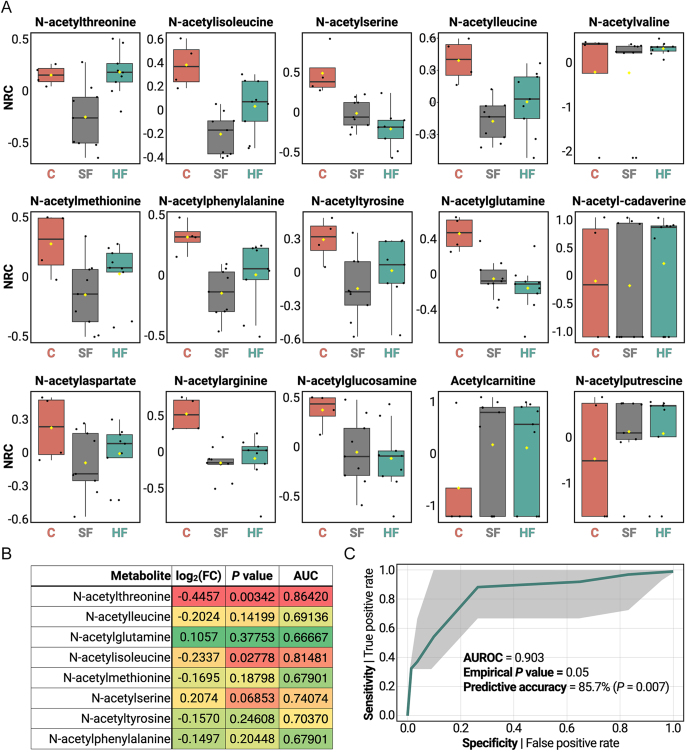
N-acetylated metabolites as discriminators of fertility status. (A) Boxplots of NRCs of acetylated metabolites across sub-fertile (SF), highly fertile (HF), and unconditioned medium control (C) groups. (B) Summary of select metabolite fold change (FC), *P*-value, and area under the receiver operating characteristic curve (AUROC) values for SF vs HF comparisons. (C) Receiver operating characteristic (ROC) curve generated using a panel of eight metabolites from (B) to classify whether acetylated biomarker abundance can determine whether embryos were derived using semen from HF or SF bulls.

Machine learning-based biomarker analysis using a panel of eight N-acetylated amino acids yielded excellent discriminatory performance. For context, biomarker AUROC values are commonly interpreted as excellent (0.9–1.0), good (0.8–0.9), fair (0.7–0.8), poor (0.6–0.7), or fail (0.5–0.6) (Xia *et al.* 2013). The selected metabolites were N-acetylthreonine, N-acetyl-leucine, N-acetylglutamine, N-acetylisoleucine, N-acetylmethionine, N-acetylserine, N-acetyltyrosine, and N-acetylphenylalanine (Fig. 5A). Although these metabolites generally did not meet conventional univariate thresholds for differential abundance individually (*P* ≤ 0.05; |FC|>1.5), their combined model achieved an ‘excellent’ AUROC of 0.903 (Fig. 5B and C), with a cross-validated accuracy of 85.7% (Fig. 5C).

## Discussion

This study investigated whether sire fertility status is reflected in the metabolic phenotype of early embryos, as assessed non-invasively through analysis of embryo-conditioned culture media. While embryos derived from HF and SF bulls exhibited largely similar global metabolic profiles, subtle but consistent differences were detectable using multivariate analytical approaches.

IVF data for these bulls have been reported previously as part of a larger study examining a range of functional, morphological, and intracellular characteristics in cryopreserved sperm from HF and SF bulls (Bernecic *et al.* 2021). No differences were detected in the cleavage rate (48 h post-insemination) or blastocyst development between groups following IVF. It is important to emphasize, however, that the categorization of bulls as HF or SF was based entirely on field fertility performance following AI – the only parameter of direct relevance to the industry – and not on IVF outcomes. The question addressed in the current study is, therefore, whether embryos derived from bulls of divergent field fertility differ in their metabolism, irrespectively of IVF performance.

The absence of differences in IVF outcomes between HF and SF bulls is consistent with the broader literature. Current sire fertility measurements do not consistently align with *in vitro* embryo production outcomes, meaning that SF sires may perform comparably to HF sires in *in vitro* production while producing fewer pregnancies in the field (Ortega *et al.* 2024). While IVF is a valuable tool for identifying bulls with severe fertility deficits, including those not detected by standard motility assessments routinely used in most AI centers (e.g. O’Callaghan *et al.* (2023) and Fernandez-Fuertes *et al.* (2017)), it is less sensitive at distinguishing bulls of average to very good field fertility – representative of the majority of AI sires, including those used here.

This may be partly because IVF bypasses the physiological challenges of sperm transport from the site of deposition (uterine body in AI) to the site of fertilization (oviduct). Consistent with this, using the same panel of bulls described here, O’Callaghan *et al.* (2021) reported that a greater proportion of embryos recovered following superovulation and AI of heifers inseminated with HF vs SF semen were at more advanced developmental stages at day 7 and that the number of accessory sperm – while highly variable – was greater for embryos derived from HF bulls, suggesting that more sperm successfully reach the fertilization site *in vivo*.

Across all treatment groups, 149 metabolites were detected, comparable to the number reported in media from day 8 bovine embryos and controls (132 and 98, respectively) in previous work (Simintiras *et al.* 2021). The predominance of amino acids (56%) and lipids (17%) among detected metabolites is consistent with the composition of standard embryo culture media and with the established importance of amino acid metabolism during preimplantation development (Takahashi & First 1992, Rosenkrans & First 1994, Keskintepe *et al.* 1995, Leese *et al.* 2021).

Unsupervised analyses revealed substantial overlap between HF and SF bull-derived embryo-conditioned media, indicating largely similar global metabolic profiles. This is not entirely unexpected, given that SF bulls are not infertile, but rather exhibit reduced fertility relative to the population mean. The variability observed among bulls within fertility groups likely reflects a combination of inherent biological variation in embryo metabolism and sire-associated factors, including differences related to semen processing or cryotolerance.

In contrast, both HF and SF bull-derived embryo-conditioned media were clearly distinct from unconditioned (control) media, reflecting active metabolic conditioning by developing embryos. This separation enabled initial characterization of general features of *in vitro* embryo metabolism before focusing on sire fertility effects. When metabolites differentially abundant in HF and SF bull-derived embryo-conditioned media vs control comparisons were considered together, 30 metabolites were consistently altered by embryo presence, irrespective of sire fertility.

Of these, 12 metabolites (3-methyl-2-oxobutyrate, 3-methyl-2-oxovalerate, 4-methyl-2-oxopentanoate, alpha-ketoglutarate, choline, creatine, hypoxanthine, ornithine, spermine, thymidine, uridine, and xanthine) were increased in embryo-conditioned media (i.e. released by embryos). Moreover, eight of these metabolites (3-methyl-2-oxobutyrate, 3-methyl-2-oxovalerate, 4-methyl-oxopentanoate, creatine, hypoxanthine, spermine, thymidine, and xanthine) were not detected in control media, suggesting that they are specifically produced by embryos.

Functionally, ornithine, spermine, and creatine are important intermediates, or by-products, of arginine metabolism and the urea cycle (Morris 2016, Wu *et al.* 2018, Bazer *et al.* 2025, Fung *et al.* 2025). Arginine and related metabolites have been previously associated with embryo viability and developmental rate in ungulates (Perkel & Madan 2017, Tsopp *et al.* 2025) and, together with the present findings, support the established notion that arginine metabolism is tightly regulated during early embryo development (Bazer *et al.* 2015, 2018). Similarly, the accumulation of branched-chain ⍺-keto acids (3-methyl-2-oxovalerate, 3-methyl-2-oxobutyrate, and 4-methyl-2-oxopentanoate) reflects branched-chain amino acid catabolism. These ⍺-keto acids can be converted to acyl-CoA derivatives that enter the tricarboxylic acid (TCA) cycle to support energy production; ⍺-ketoglutarate participates in these reactions as an amino group acceptor, facilitating the conversion of amino acids to ⍺-keto acids and glutamate (Neinast *et al.* 2019, Mailloux 2024). Collectively, these patterns are consistent with robust amino acid metabolism in early mammalian embryos (Partridge & Leese 1996, Van Winkle 2001). Whether these metabolites primarily reflect intracellular metabolic flux (i.e. waste products) or also contribute to extracellular homeostasis (e.g. maintaining osmotic balance) remains an open question.

Conversely, 18 metabolites (2-hydroxyglutarate, 2-methylcitrate/homocitrate, 2-oxoarginine, 3-hydroxyadipate, 5-oxoproline, aspartate, (iso)butyrate, caproate, cystine, glutamine degradant, isovalerate, kynurenine, maleate, myo-inositol, N-acetylarginine, phosphate, triethanolamine, and valerate) were consistently decreased in the presence of embryos, suggesting preferential uptake and utilization. Valerate, caproate, (iso)butyrate, and isovalerate are short-chain fatty acids that can be oxidized to acyl-CoA intermediates and enter the TCA cycle (Szrok-Jurga *et al.* 2023), again highlighting central carbon metabolism as a major energetic hub. Additional depleted metabolites, including N-acetylarginine, aspartate, and 2-oxoarginine, are also involved in arginine metabolism and the urea cycle, further underscoring the centrality of this pathway in early development. When statistical significance (*P* ≤ 0.05) was considered without a FC threshold, an additional 39 metabolites were affected by embryo presence, indicating that more than half of all detected metabolites were either consumed or released by embryos during culture. This highlights the breadth of metabolic activity occurring during early development.

When HF and SF bull-derived embryo-conditioned media were compared directly, only three metabolites (2-piperidinone, N-lactoyl leucine, and N-succinyl leucine) met the combined criteria for differential abundance (*P* ≤ 0.05; |FC|>1.5). While these metabolites have not been characterized in the context of sperm or embryos, N-lactoyl amino acids are increasingly recognized as signaling molecules and potential biomarkers of metabolic and pathological states (Naja *et al.* 2025). N-lactoyl leucine has also been proposed as a more bioavailable alternative to leucine in culture media (Schmidt *et al.* 2021); however, its increased abundance in HF vs SF bull-derived embryo-conditioned media is more consistent with reduced uptake than enhanced bioavailability, and its potential functional relevance in this context remains unclear.

Overall, the limited number and modest magnitude of HF vs SF bull-derived embryo-conditioned medium differences suggest that sire fertility status exerts only a subtle influence on the gross metabolic profile of resulting early embryos under these conditions. This interpretation is consistent with previous findings from the same bull cohort, which reported no differences in fertilization rate or blastocyst yield between HF and SF bulls (Bernecic *et al.* 2021). This suggests that field fertility is not strongly determined by early embryo metabolism or blastocyst formation *per se*.

Metabolic profiling was restricted to embryos that successfully developed to the blastocyst stage. Consequently, the analysis only captures the metabolism of developmentally competent embryos and may not reflect variation in embryos that arrest prior to this. It is plausible that more pronounced sire-associated differences would be detectable if conditioned culture media were collected from continuous cultures encompassing embryos across a range of developmental outcomes. Future studies profiling conditioned embryo culture media at multiple time points across the culture period and including embryos that fail to reach the blastocyst stage will help clarify whether sire-influenced metabolic differences emerge earlier in development and, therefore, whether they are predictive of subsequent embryo viability.

A limitation of the present study is the small number of bulls per fertility group. Intra-bull variation in embryo metabolism may contribute to the overlap observed between HF and SF groups in unsupervised analyses. Future studies employing mixed-effects models or bull-level averaging, while incorporating a larger number of sires, would provide more robust estimates of the paternal effect on embryo metabolic phenotype.

Nonetheless, given the absence of strong univariate associations between bull fertility and embryo metabolism, we next applied machine learning approaches to identify multivariate, non-invasive metabolic biomarker panels of bull fertility. Notably, a panel of eight N-acetylated amino acids discriminated embryos according to sire fertility status with high predictive accuracy. N-acetylation converts free amines into amides, generally reducing protein and amino acid reactivity and increasing molecular stability (van de Kooij *et al.* 2023). The enrichment of N-acetylated amino acids in the biomarker panel may reflect the extensive amino acid turnover in embryos, with preferential export of more stable, N-acetylated, species potentially contributing to osmotic regulation or detoxification of excess amino acids. Importantly, most of these metabolites did not meet conventional univariate thresholds for differential abundance, yet collectively achieved excellent classification performance, consistent with the principle that predictive biomarkers need not be strongly or individually ‘significant’ (Lo *et al.* 2015). Validation in larger independent bull populations will be required to assess the robustness and generalizability of this biomarker panel.

Numerous studies have investigated differences between HF and SF bulls, including analyses of sperm composition (Donnellan *et al.* 2022, Rabaglino *et al.* 2022, Hoorn *et al.* 2025) and downstream effects on early pregnancy (O’Callaghan *et al.* 2021). In particular, O’Callaghan *et al.* (2021) reported increased conceptus (embryo and extraembryonic tissues) recovery following insemination with HF vs SF semen, indicating that sire fertility can influence early conceptus competence. The present findings suggest that such differences are not mirrored by large-scale shifts in early embryo metabolism detectable in culture media. Nonetheless, subtle metabolic differences established during the preimplantation stage could have disproportionate downstream consequences for conceptus development and survival. From an applied perspective, the machine learning framework described here provides a non-invasive, metabolomics-based approach for classifying embryos based on sire fertility status with high accuracy. Given the complexity of sperm biology and embryogenesis, however, a comprehensive understanding of paternal influences on pregnancy outcome will likely require integration of multiple data layers – including sperm multi-omics, embryo phenotyping, and invasive intracellular embryo metabolic profiling – to fully resolve the mechanisms linking sire fertility to subsequent embryo viability.

## Supplementary materials







## Declaration of interest

The authors declare that there is no conflict of interest that could be perceived as prejudicing the impartiality of the work reported.

## Funding

This work was supported by Science Foundation Ireland (16/IA/4474) (DAK, SF, and PL); the European Union Marie Skłodowska-Curie Action Doctoral Network (101120104) (QH); the State of Louisiana Board of Regents (LEQSF(2023-26)-RD-A-03) (CAS); the Audubon Center for Research of Endangered Species (GR-00013800) (CAS); the Joe W and Dorothy Dorsett Brown Foundation (BG008584) (CAS); and the United States Department of Agriculture (USDA) Research Capacity (Hatch) funds (LAB-94578) (CAS).

## Author contribution statement

PL, SF, DAK, and CAS contributed to the experimental design. QH, EOC, PL, and CAS performed experimentation and data analysis. QH and CAS wrote the manuscript. All authors revised and approved the final manuscript.

## Data availability

All relevant data are provided as supplementary material.

## Ethical approval

Semen samples were obtained as surplus material from routine collections at commercial AI centers in Ireland. All other biological materials were collected post-mortem from commercial abattoirs. No procedures were performed on live animals; therefore, formal ethical approval was not required.
